# Hernia—Surgical Treatment and Radical Cure*Kansas State Homœopathic Society, 1896.

**Published:** 1896-07

**Authors:** William Davis Foster

**Affiliations:** Professor of Surgery, Kansas City Homœopathic Medical College


### HERNIA—SURGICAL TREATMENT AND RADICAL CURE.*

WILLIAM DAVIS FOSTER, M. D., PROFESSOR OF SURGERY, KANSAS CITY HOMŒOPATHIC MEDICAL COLLEGE.

Most of the operations for the radical cure of hernia are so new that the surgical world is only beginning to grasp their vast importance. Operations for the radical cure of hernia will within the next decade, I venture to predict, become so common that an impress will be left upon this period as an epoch in surgery of no less distinction than that made for kolpohysterectomy by Czerny and others within the decade now closed.

When once the utility of this subject is fully realized by surgeons the popularization of the operation will quickly follow. Of the six or seven millions of persons in the United States who have hernia at the present time, the numbers will so rapidly diminish that the next census will doubtless show a marked change in this respect.

A brief inquiry into the history of operations for the surgical cure of hernia discloses the fact that very little was done until within about twenty-five years. Writers previous to that time have generally ignored the subject or treated the proposed operations with contempt or suspicion. The nestor of American surgeons, the late Samuel D. Gross, fails to record one word in mention of any operation having this end in view. In the early part of the present century, Langenbeck, Schumacker, the Coopers, Warren, and other surgeons, made operations for the radical cure with success. It is also certain that the operation quickly fell into disrepute, and was wholly lost sight of until about 1870, when Lister made known to the world his antiseptic views, which caused a new impulse to surgery in every department.

---

* Kansas State Homœopathic Society, 1896.

Operations for herniotomy are well known. They are historic, and it strikes the modern surgeon as singular that the older operators should make a herniotomy without resorting to any method to accomplish a radical cure. The fact is that herniotomy in time past fell into disrepute in consequence of the high rate of mortality following the operation. In this respect a parallel process of reasoning condemned, not many years before, the operation of trephining the cranium for gunshot and other injuries. It is stated in the *Medical and Surgical History of the War of the Rebellion* that the mortality rate following trephining in some of the wars of Napoleon was so great that its practice was interdicted by Baron Larry and others. A late writer, in discussing this question, justly observes that the high death-rate following the operation of trephining was not the result of the operation itself, but that death resulted from the injury which rendered the trephining necessary. It is equally true that most of the deaths which have followed the operation for herniotomy have resulted, not from the operation itself, but from the fact that the condition of strangulation, which rendered the operation necessary, had already produced such pathological conditions as to render death inevitable.

Surgeons of the present day take note of these facts, and operate early before tissue changes to any great extent have occurred, and then follow the release of the strangulation by some of the varied methods for radical cure. Different operators for radical cure practice and advocate different methods. The object to be attained is the same by all. Whatever method secures the desired result must certainly be commendable. Statistics show that death from selected operations for the radical cure of hernia are extremely rare, that when the operation is done properly under aseptic conditions, the cure is practically certain.

There is a notion prevalent, not only amongst professional people, but also widespread amongst the laity, that operations for hernia are not only dangerous to life, but frequently fail to cure the rupture. Nothing could be more erroneous. It is perfectly certain that there is more menace to life from the hernia itself than there is from the surgical operation for the cure of hernia. This is true in general. It is emphatically true in regard to femoral hernia. This form of rupture is mostly found in women. The canal through which the hernia escapes, the crural ring, is extremely small. In case the hernia is composed of intestine, the dangers of strangulation are very great, and death is very rapid, the patient even going into collapse, and passing beyond surgical remedy before a decision to operate has been made, or before the surgeon can reach the patient. The day is near at hand when surgeons will advise and the people will be ready to accept operations for a radical cure in every case where a cure by other means fails. I am not quite certain in my own mind whether it would not be better in cases of congenital hernia, even, to resort to an operation for radical cure, in preference to the uncertain, and often futile attempt to cure by the application of trusses and otherwise.

This question is of such importance that its free discussion by surgeons should be encouraged, and the results of operations for radical cure widely disseminated, so that the prevalent errors may be speedily abandoned.

